# Assessing the psychometric and ecometric properties of neighborhood scales using adolescent survey data from urban and rural Scotland

**DOI:** 10.1186/s12963-017-0129-1

**Published:** 2017-03-28

**Authors:** Gina Martin, Joanna Inchley, Gerry Humphris, Candace Currie

**Affiliations:** 1 Child and Adolescent Health Research Unit, School of Medicine, Medical and Biological Sciences Building, University of St Andrews, St Andrews, KY16 9TF UK; 2 School of Medicine, Medical and Biological Sciences Building, University of St Andrews, St Andrews, KY16 9TF UK

**Keywords:** Neighborhood, Ecometrics, Urban, Rural, Adolescence, Factor analysis, Invariance

## Abstract

**Background:**

Despite the well-established need for specific measurement instruments to examine the relationship between neighborhood conditions and adolescent well-being outcomes, few studies have developed scales to measure features of the neighborhoods in which adolescents reside. Moreover, measures of neighborhood features may be operationalised differently by adolescents living in different levels of urban/rurality. This has not been addressed in previous studies. The objectives of this study were to: 1) establish instruments to measure adolescent neighborhood features at both the individual and neighborhood level, 2) assess their psychometric and ecometric properties, 3) test for invariance by urban/rurality, and 4) generate neighborhood level scores for use in further analysis.

**Methods:**

Data were from the Scottish 2010 Health Behaviour in School-aged Children Survey, which included an over-sample of rural adolescents. The survey responses of interest came from questions designed to capture different facets of the local area in which each respondent resided. Intermediate data zones were used as proxies for neighborhoods. Internal consistency was evaluated by Cronbach’s alpha. Invariance was examined using confirmatory factor analysis. Multilevel models were used to estimate ecometric properties and generate neighborhood scores.

**Results:**

Two constructs labeled neighborhood social cohesion and neighborhood disorder were identified. Adjustment was made to the originally specified model to improve model fit and measures of invariance. At the individual level, reliability was .760 for social cohesion and .765 for disorder, and between .524 and .571 for both constructs at the neighborhood level. Individuals in rural areas experienced greater neighborhood social cohesion and lower levels of neighborhood disorder compared with those in urban areas.

**Conclusion:**

The scales are appropriate for measuring neighborhood characteristics experienced by adolescents across urban and rural Scotland, and can be used in future studies of neighborhoods and health. However, trade-offs between neighborhood sample size and reliability must be considered.

## Background

The impact of neighborhood conditions on health and well-being outcomes has been gaining considerable attention over the past decade [1]. Young people may be especially affected by the neighborhood they live in due to limited mobility restricting each individual’s school, family, and peers to a confined geographic area [2, 3]. Many studies have explored the impact of neighborhood social conditions on adolescent health outcomes including self-rated health (e.g., [2]), alcohol use (e.g., [3]), and violence (e.g., [4]). In line with this increased research interest, there is a need for measurement instruments that examine the features of the neighborhood in order to better understand the relationships between the neighborhood context and adolescent health and well-being [5–7]. Despite this, there are few validated and reliable measures of adolescent neighborhood conditions [6], particularly at the neighborhood level.

Most studies examining neighborhood level conditions make use of structural measures which are based on administrative data such as census information. Recently research has moved beyond examining the structural features of the neighborhood to better understand the societal conditions present at the neighborhood level. Survey data have proven to be a useful source in understanding the social conditions of the neighborhoods in which people reside [5, 8]. However, many studies survey adults to understand the neighborhoods in which the adolescents live, leaving adolescents ignored as active agents within their own neighborhoods [9, 10]. Schaefer-McDaniel [11] argues that this represents a methodological flaw and that adults cannot fully represent with accuracy the experiences and perceptions of young people in their environment. Measures derived from adolescents’ perspectives are therefore considered more theoretically valid than adult measures of the adolescent environment, as young people may have different perceptions of their neighborhood than adults, are exposed to fewer neighborhoods to compare their own with, and access different areas of their neighborhood [12].

Neighborhoods are experienced through an individual’s perceptions and as a collective attribute at an aggregate level (a shared characteristic). Where possible, examining both collective measures and individual perceptions is desirable to allow for the most complete picture of the role of neighborhoods in adolescents’ lives. Kawachi et al. [13] argue that studies examining the relationship between neighborhood social conditions and health should consider both individual perceptions and collective conditions using multilevel frameworks and considering cross-level interactions. For instance, socially isolated individuals may still benefit from residing within a community with positive neighborhood conditions. The construction of valid and reliable measures that operate at both the individual and neighborhood level necessitates an assessment of both psychometric and ecometric properties. Psychometric properties refer to the extent to which items reliably capture a construct at the individual level, while ecometric properties refer to the reliability at the neighborhood level [5]. Although some studies exist detailing the psychometric properties of adolescents’ neighborhood perceptions (e.g., [6]) fewer studies examine the ecometric properties of these measures [12].

An important consideration when deriving neighborhood scales that will be utilized in a variety of neighborhood settings is whether the scale items are operationalized similarly for different types of regions, and what adaptations might be needed to ensure scales are appropriate across neighborhood types. The same scales therefore may not be invariant between urban and rural areas [14]. Neighborhood scales are considered invariant when items within the scale function similarly between different groups (see [15, 16] for a more complete discussion). This makes comparisons between groups justifiable. Two types of invariance are most frequently considered 1) factor loading invariance (*metric* invariance) and 2) intercept invariance (*structural* invariance). Metric invariance indicates the factor loadings are equal across groups; if this condition is met, “weak” invariance is satisfied [17]. Reasons metric invariance may not be met include: if respondents from different groups interpret the scale items differently or if certain groups have a higher propensity to extreme responses [16]. Structural invariance indicates that a one-unit change in the item response results in the same change on the underlying factor for both groups. This meets the condition for “strong” invariance [17]. Structural invariance may not be met if certain groups have a different reference point when making statements about themselves, there are differences in social norms, and/or certain groups are prone to respond strongly to an item despite having comparable factor values [16, 18]. Structural invariance implies both the meaning of constructs and levels of the underlying items are the same between groups; thus allowing for group comparisons [19].

This research seeks to construct multi-item scale(s) measuring adolescent’s social environment in the neighborhoods in which they live. Accordingly, both individual and neighborhood measures are derived from adolescent survey data. Psychometric methods are used to validate and measure reliability of individual level measures while ecometric methods are used to measure reliability at the neighborhood level [20]. It is important to have both valid and reliable measurements prior to conducting statistical models using these constructs. Accordingly, the objectives of this research were to: a) establish valid and reliable measures of adolescent neighborhood conditions, b) assess the psychometric and ecometric properties of these measures, c) test for invariance between urban/rural classifications, and d) generate neighborhood level scores that can be used in further analysis.

## Methods

### Study population, study questionnaire, and data

This research utilizes data from the Scottish 2010 Health Behaviour in School-aged Children (HBSC) survey, a World Health Organization (WHO) collaborative cross-national study conducted in 44 countries in Europe and North America [21]. The anonymous questionnaire was paper-based and completed in-class under teacher supervision. The data are a nationally representative sample of pupils in Secondary 4 (S4), age approximately 15.5 years, that also includes a boost of rural schools [22] (*n* = 3591).

Students supplied their residential postcode which was used to determine their location of residence and their urban/rural status. Urbanity was classified into six categories based on the urban-rural postcode classifications by the Scottish Government: 1) *4 cities* (settlements with population over, 125,000: Aberdeen, Dundee, Glasgow, and Edinburgh) (*n* = 620), 2) *other urban* (other settlements with a population over 10,000) (*n* = 617), 3) *accessible towns* (settlements with a population between 3000 and 10,000 and within a 30-min drive time of a settlement of 10,000 or more) (*n* = 274), 4) *remote towns* (settlements with a population between 3000 and 10,000 and more than a 30-min drive time of a settlement of 10,000 or more) (*n* = 247), 5) *accessible rural* (settlements with a population <3000 and within a 30-min drive time of a settlement of 10,000) (*n* = 376) & 6) *remote rural* (settlements with a population <3000 and more than a 30-min drive time of a settlement of 10,000 or more) (*n* = 456). “Neighborhoods” were represented by Intermediate Data Zones (IDZs). IDZs were developed by the Scottish Government. These zones represent 1235 regions in Scotland, contain on average 4000 residents and are based on administrative data and local knowledge [23].

A set of indicators of neighborhood conditions were previously developed by the HBSC international network, a multinational group of experts in the field of adolescent health (Table 1). Prior to data collection the neighborhood conditions questions were piloted in several countries including Scotland to ensure adolescents understand the meaning of the questions [24]. The goal of many of these indicators was to measure neighborhood social capital specifically for young people drawing on multiple theoretical perspectives [25] and they were based partially on social capital measures used by Kawachi et al. [26] and on qualitative analysis undertaken by Morrow [27]. Other items addressing neighborhood conditions were included in the current analysis regarding neighborhood safety, general perception of neighborhood and presence of certain behaviors and physical features (e.g., rundown buildings). One item regarding the local area was not included in this analysis: “How well off is the area in which you live?” This exclusion was made because this item assessed economic conditions rather than social environment. It did not, therefore, fit theoretically with the other items. These items have been used in multiple past studies either in their entirety, or using a subset (e.g., [28–30]). Items were recoded so that higher values indicated greater presence of each item.

**Table 1:** HBSC questions regarding local area social environment and factors loadings of HBSC items regarding the neighborhood social environment (*n* = 3396)

Item number	Item	Value range	Factor 1	Factor 2
1	Feel safe in local area	1 “always”- 4 “rarely or never”	.319	−.397
2	Local area is a good place to live	1 “yes, it is really good”-5 “no, it is not good at all”	.423	−.352
3	In the area where you live you can trust people around here	1 “agree”—5 “disagree a lot”	**.691**	
4	People say “hello” and talk to each other in the streets in the area where you live	1 “agree”—5 “disagree a lot”	**.665**	
5	It is safe for younger children to play outside in the area where you live	1 “agree”—5 “disagree a lot”	**.596**	
6	There are good places to spend free time in the area where you live	1 “agree”—5 “disagree a lot”	.397	
7	I could ask for help or favour from a neighbour in the area where you live	1 “agree”—5 “disagree a lot”	**.675**	
8	Most people around here would try to take advantage of you if they got a chance in the area where you live	1 “agree”—5 “disagree a lot”		.385
9	In the area where you live are there are groups of young people who cause trouble	1 “lots”- 3 “none”		**.809**
10	In the area where you live are there are litter, broken glass or rubbish lying around	1 “lots”- 3 “none”		**.769**
11	In the area where you live are there are run-down houses or buildings	1 “lots”- 3 “none”		**.619**
Eigenvalue			4.30	1.47

Factor loadings below .30 are not reported

Bold indicates the item loaded above .40 on a factor and did not cross-load

### Analysis

#### Exploratory factor analysis

As a first step exploratory factor analysis was conducted examining the structure of latent variables derived from the items in Table 1. The number of respondents with complete data on all questions of interest was 3396 out of 3591. Two factors were decided on based on the scree plot and the retaining all factors with an eigenvalue of greater than 1.0 [30]. As suggested by Costello and Osborne [31], an oblique rotation was utilized and direct oblimin extraction was conducted by principal axis factoring. Items were retained if they had a factor loading > .40 and did not cross-load on another factor (factor loading > .32, which equates to approximately 10% overlapping variance with other items in that factor) [31, 32]. Psychometric properties of each scale were assessed using Cronbach’s alpha coefficient [33].

#### Confirmatory factor analysis and invariance testing

Secondly, a confirmatory factor analysis (CFA) was conducted to determine whether the proposed latent variables exhibit equivalence across urban and rural settings using measurement invariance testing methods. This analysis was limited to a subset of the total sample which included those with valid residential postcode data allowing for classification of residential urban or rural conditions (*n* = 2590). Those excluded due to missing postcode data had a higher proportion of males compared to those who reported their postcode (53% versus 47%; Chi-Square =10.5; *p* < .01) but were not significantly different from those who reported their postcode in terms of the HBSC family affluence scale [20].

As noted by Bryne [34], testing for invariance requires a series of hierarchical steps (Table 2). First, a *configural* model was run (a model where no constraints are placed between groups but the data for all groups are analyzed simultaneously). This model acts as the baseline. Secondly, a *metric* model was established where factor loadings were constrained to be equal among groups. This assesses metric invariance. Third, a *structural* model was run where the factor loadings and intercepts are constrained to be equal. This model was compared to the metric model to assess for “strong” invariance. Because there is debate in the literature regarding how best to test for invariance each model is compared to the subsequent model using four tests: 1) a chi-square (*X* ^*2*^) difference test where a nonsignificant value indicates invariance [34], 2) the ratio of the change in *X* ^*2*^ to the change in degrees of freedom between two models (∆*X* ^*2*^/∆df) where a value ≤ 5 indicates invariance [15, 35], 3) the difference in root mean square error of approximation (RMSEA), and 4) comparative fit index (CFI) values, where a difference ≤0.015 and ≤ .01 respectively indicate invariance [34, 36, 37].

**Table 2:** Description of measurement invariance

Invariance type	Description
Configural	Different groups associate the same subset of items with the same constructs. To test data are analyzed simultaneously and no constraints are placed between groups. This model is used as the baseline model.
Metric (also called weak invariance)	Respondents across groups attribute the same meaning (factor loadings) to the latent construct(s). To test factor loadings are constrained to be equal across groups. This model is compared to the configural model.
Structural (also called scalar or strong invariance)	The meanings (factor loadings) and the levels of the items (intercepts) are equal across groups. To test factor loadings and intercepts are constrained to be equal. This model is compared to the metric model. If this is met groups can be compared on scores of the latent construct.

From [16, 18, 32, 34]

#### Ecometrics

Ecometric approaches were used to derive neighborhood scores and to test the reliability of the neighborhood measure using linear three-level models [5, 20, 38–40]. The question response is the dependent variable, level one is a categorical variable of the question/item, level two is the individual, and level three is the neighborhood. The reliability of the neighborhood level measure was calculated as a function of the neighborhood variation and the neighborhood sample size. The value is close to 1 when the neighborhood means vary substantially across neighborhoods (holding sample size constant) or the sample size per group is large [41]. Although there is no agreed cut-off for reliability at the neighborhood level, generally scores above 0.60 are considered good or acceptable [42, 43]. Ecometrics mitigates issues associated with using scale means to aggregate to the neighborhood level because it takes individual differences in perceived neighborhood social environment into account by including these as level-two covariates. Measures therefore reflect differences by geographic area rather than respondents’ individual characteristics therefore controlling for possible measurement bias [43]. The residuals are used as the neighborhood variable because they represent what cannot be attributed to individual response patterns with positive values reflecting higher than average levels [39]. It is important to bear in mind that group level coefficients represent a weighted average estimate of each grouping towards the average of the dataset based on group sample size and distance between the group level estimate and the overall estimate, termed “*shrinkage,*” thus potentially biasing the estimates towards the overall estimate [43]. Although some research refutes the value of the added complexity of ecometrics over simple mean aggregation as the results are similar [44], ecometrics allows for reliability to be calculated which is an important aspect of scale development. Reliability is calculated based on Hox [43].

$$ Reliability=\kern0.5em \frac{neighbourhood\  variance}{neighbourhood\  variance + \left(\frac{individual\  variance}{\begin{array}{c}\hfill average\  number\ \hfill \\ {}\hfill of\  people\  per\  area\hfill \end{array}}\right)+\left(\frac{item\  variance}{\begin{array}{c}\hfill number\ast average\  number\ \hfill \\ {}\hfill \kern0.5em  of\  items\kern1em  of\  people\  per\  area\hfill \end{array}}\right)\ } $$

Individual item responses were imputed prior to ecometric analysis using the person average of available items in each scale, if less than half were missing [45]. Imputation methods on item responses have been used in similar models [46]; alternatively, as stated by Hox [43], the model can accommodate missing data. However, the best approach to missing items in these types of models is still under study [46].

Individuals residing in an area with less than five respondents were excluded. This cut-off is similar to other studies of adolescent neighborhoods [39]. We also conducted a sensitivity analysis where the cut-off was four, as this would allow for additional IDZs to be included in the analyses. Additionally those who were missing data on any of the scale items after imputation (four individuals on each scale had imputation procedures) were excluded, leaving 1491 respondents on the neighborhood disorder scale and 1509 on the social cohesion scale from approximately 190 IDZs for both scales. Those included did not have a significantly higher proportion of males than those not included from the total sample but they were significantly more likely to be in the high family affluence tertile (38% versus 33%). Respondents’ sex was adjusted for in the model as it may influence individual experiences of their neighborhood [39].

## Results

### Exploratory factor analysis

The scree plot indicated a two-factor solution explaining 42.7% of the variance (34.3% in the 1st factor and 8.4% in the 2nd factor).

Using a two-factor solution, the factors are 1) social cohesion (Items 3, 4, 5, 7), and 2) neighborhood disorder (Items 9, 10, 11). “Perceived good places,” “feeling safe,” and “people would try to take advantage of you” did not load > .4 on either factor while perceiving the local area as good cross-loaded between the two factors (see Table 1). A three-factor solution was also obtained and yielded similar results with the exception that “having good places to spend free time” loaded on its own factor. Given current debate on best methods to determine the number of factors to maintain, we also conducted a parallel analysis with Velicer’s minimum average partial criteria using an R-add on designed for SPSS [44]. The Velicer’s minimum average partial criteria indicated two factors be maintained and the parallel analysis indicated four factors. A four-factor solution produced two non-trivial factors that were similar to the two-factor solution presented earlier. Therefore, a two-factor solution was implemented in the CFA. Cronbach’s alpha for social cohesion was .787 and the alpha for neighborhood disorder was .765.

### Confirmatory factor analysis

From the EFA results, a two-factor solution was specified using AMOS software, using maximum likelihood (ML) estimation. Results of the *configural* model indicated good model fit (RMSEA = .027, goodness of fit index (GFI) = .975, CFI = .970, Tucker-Lewis Index (TLI) = .951). However, *X* ^*2*^ difference tests indicated non-invariance (difference in *X* ^*2*^ was significant between the *configural* model and *metric* model as well as between the *metric* model and the *structural* model) while ∆*X*2/∆df, RMSEA, and CFI difference tests indicated invariance between model comparisons (Table 3). Results of the modification indices were examined and Item 7 (“I could ask for help or favour from a neighbour”) was removed due to high error covariance with other items. Removing this item from the social cohesion scale yielded a Cronbach’s alpha of .760.

**Table 3:** Model fit statistics for invariance testing for seven- and six-item models (*n* = 2590)

Model	*X* ^*2*^	∆*X* ^*2*^	df	∆df	(∆*X* ^*2*^/∆df)	RMSEA	∆RMSEA	CFI	∆CFI
*Seven-item model*									
Configural	228.63		78			.027		.970	
Metric	277.28	48.65^a^	103	25	**1.95**	.026	**−.003**	.965	**−.005**
Structural	308.76	31.48^a^	118	15	**2.10**	.025	**−.001**	.962	**−.003**
*Six-item model*									
Configural	106.76		48			.022		.986	
Metric	130.79	**24.03**	68	20	**1.20**	.019	**−.003**	.985	**−.001**
Structural	166.49	35.70^a^	83	15	**2.38**	.020	**.001**	.980	**−.005**

^a^significant at 0.05

Bolded values indicate invariance

After removing Item 7, the six-item *configural* model indicated improved model fit (RMSEA = .022, GFI = .986, CFI = .986, TLI = .973), meeting the requirement for *configural* invariance. Additionally criteria for *metric* invariance were met by all four tests; *structural* invariance was met using the ∆*X*2/∆df, RMSEA, and CFI tests (Table 3).^1^

There were significant differences between urban and rural areas on both perceived neighborhood social cohesion and perceived neighborhood disorder found through analysis of variance (ANOVA) tests (Table 4). On average, adolescents in rural areas perceived greater social cohesion and lower neighborhood disorder in their local area; whereas individuals living in accessible small towns perceived the greatest neighborhood disorder.

**Table 4:** Mean individual perceived neighborhood social cohesion (range 3–15) and perceived neighborhood disorder (range 3–9), *n* = 2590 (95% confidence intervals)

	Social cohesion	Neighborhood disorder
Large urban areas	11.05 (10.83,11.26)	5.03 (4.90, 5.15)
Other urban areas	11.34 (11.13,11.55)	4.98 (4.85, 5.11)
Accessible small town	11.75 (11.47,12.04)	5.39 (5.22, 5.56)
Remote small town	11.99 (11.69, 12.29)	4.93 (4.74, 5.12)
Accessible rural	12.53 (12.28,12.78)	4.53 (4.38,4.67)
Remote rural	12.75 (12.54, 12.97)	4.60 (4.47, 4.72)

### Ecometrics

The ecometric properties of both neighborhood level social cohesion and neighborhood level disorder are shown below. Both scales showed moderate reliability, but within the range considered acceptable in several other studies, at .577 and .563 respectively [39, 41, 47].

$$ Reliabilit{y}_{SC} = 0.577 = \frac{.1101}{.1101 + \left(\frac{.4376}{7.7784}\right)+\left(\frac{.5685}{\left(3\ast 7.7784\right)}\right)\ } $$

$$ R e l i a b i l i t{y}_{ND}=0.563=\frac{.0367}{.0367+\left(\frac{.1585}{7.8063}\right)+\left(\frac{.1920}{\left(3\ast 7.8063\right)}\right)} $$

Sensitivity analysis showed that when the cut-off was changed to four individuals per IDZ rather than five per IDZ, the reliability for neighborhood social cohesion and neighborhood disorder dropped to .524 and .543, respectively. However, the number of neighborhoods increased from approximately 190 to 250. Additionally, the number of individual survey respondents increased by approximately 250. Given the substantial increase in neighborhoods and that the reduction in reliability was not great (reliability was still > .50) we proceeded with analysis using the cut-off of four (IDZs *n* = approximately 250). Moreover, due to the small number of response categories in the neighborhood disorder items, we re-ran the original model as an ordinal three-level model. This also made little difference to reliability (reliability = .589 versus 0.563).

Convergent validity was tested by examining the correlations between neighborhood level constructs and administrative measures available for the IDZs from the Scottish Government [48]. We examined the percent of people living within 500 m of a derelict site in 2010 expecting to find a positive correlation with neighborhood disorder, as well as the estimated percent of working aged households that were materially deprived in 2008/2009 expecting to find a negative correlation with neighborhood social cohesion, as has been found in past studies using adult survey measures [49]. As was expected, neighborhood social cohesion and neighborhood disorder were significantly and negatively correlated (*R* =−.499, *p* < .001). Also, a positive correlation was found between proportion of people living near derelict sites and neighborhood disorder (*R* = .365, *p* < .001) and a negative association was found with neighborhood social cohesion (*R* =−.320, *p* < .001). In terms of material deprivation, a negative correlation was present with neighborhood social cohesion (*R* =−.396, *p* < .001) and a positive correlation was found with neighborhood disorder (*R* = .410, *p* < .001).

## Discussion

To our knowledge, this is the first attempt to construct neighborhood scales for adolescents at both the individual and neighborhood level that takes into account potential invariance across neighborhood type. Measures across two dimensions of adolescents’ neighborhood social environment were constructed with both yielding good reliability at the individual level and moderate reliability at the neighborhood level. However, it is important to note that the response system varied for the neighborhood questions and that the EFA results largely corresponded with this. Nevertheless, the two measures perform well in CFA.

The research findings of this study are consistent with past research of psychometric and ecometric properties of adolescent neighborhood scales. Studies of rural and urban United States adolescents found similar individual level reliabilities. For example, a measure of neighborhood attachment that used some similar indicators to this study reported a Cronbach’s alpha of .72 [50] and a measure of neighborhood deterioration using comparable measures reported a Cronbach’s alpha of .75 [4]. Additionally, findings are consistent with a study of neighborhood level social capital in Dutch adolescents which found what the authors deem acceptable levels of neighborhood social capital at .57 [39].

We found that adjustments to the originally specified model improved model fit and measures of invariance. The results of invariance testing indicate “weak” (*metric*) invariance between different urban/rural locations for the six-item model was certainly met. There is also evidence of “strong” (*structural*) invariance, however, these results are more sensitive to estimation procedure and invariance test used and therefore should be interpreted with caution. Issues with *X* ^*2*^ difference test have been widely noted as it is sensitive to sample size [15, 34, 36]. Therefore the other approaches used to test for invariance may be more appropriate and we can be reasonably confident that strong invariance is met.

Regarding the ecometric analysis, we were able to construct measures that reflect collective attributes that showed moderate reliability. Trade-offs between neighborhood sample size and reliability had to be considered as reliability decreases as a function of within neighborhood sample size. There is no established cut-offs for reliability in ecometric analysis and so the researcher must consider the trade-off between sample size and reliability as well as the purpose of the scales prior to use in future analysis. Estimates of convergent validity were as expected indicating that valid measures of the neighborhood level social environment can be constructed using survey data from adolescents. This is similar to findings based on surveys of adults [5].

A potential limitation of the current study is that an administrative definition was used as a proxy of neighborhoods. The IDZs were constructed with consultation from those with local knowledge (by consultation with Community Planning Partnerships who coordinated the views of local people and regional officials); however, these partnerships are administratively based and therefore do not necessarily include adolescents. Additionally, the questions in the HBSC survey asked adolescents about their “local area” in which they lived but did not specify how local area should be defined and we were unable to determine how the administrative boundaries relate to the adolescents’ perceptions of their local area boundaries. This may contribute to within neighborhood variability [5]. Despite these limitations, IDZs reflect a neighborhood definition for which other data from government sources can be linked.

Another consideration when interpreting the results is the potential for bias due to the presence of missing cases; particularly the proportion who were missing due to non-reporting of postcode data and missing data due to a low number of respondents within neighborhoods. This is a common issue in studies that collect neighborhood data but are not able to target at the neighborhood level, such as in school-based surveys (e.g., [47]).

Although the measures established in this research are suitable for individuals experiencing urban and rural conditions in Scotland they may not be invariant cross-culturally. Further studies are needed to better understand how perceptions of neighborhoods may vary between countries. This represents an important avenue for future research of neighborhood characteristics. Additionally, the compromise between reliability, sample size, and having an appropriate number of respondents per neighborhood is an important area for future research.

## Conclusions

It is important that studies examining adolescent outcomes make clear whether associations are found at the individual or collective level as these indicate distinct levels at which to target potential policies and interventions, e.g., people or places [12, 51, 52]. Constructing valid and reliable measures at these different levels represents a crucial first step in understanding the ways in which adolescents experience their local areas. The two scales established in this study can be used to investigate the effect of neighborhood environmental characteristics, specifically social cohesion and neighborhood disorder, on a range of outcomes and from a population health perspective. By accessing adolescent’s own perceptions of the area in which they live, these instruments represent a more useful and appropriate means to measure the impact of neighborhood on adolescent outcomes than many existing measures which are mainly based on adult perceptions or structural indicators.

## Abbreviations

ADF: Asymptotically Distribution-free
ANOVA: Analysis of variance
CFA: Confirmatory factor analysis
CFI: Comparative fit index
DF: Degrees of freedom
EFA: Exploratory factor analysis
GFI: Goodness of fit index
HBSC: Health Behaviour of School Aged Children
IDZ: Intermediate Data Zones
ML: Maximum likelihood
RMSEA: Root mean square error of approximation
S4: Secondary 4
TLI: Tucker-Lewis Index
WHO: World Health Organization

## Footnotes

1 Ad hoc tests for multivariate normality were conducted for each urban/rurality type. Overall, the Mardia’s coefficient of multivariate kurtosis suggests non-normality in the sample (range: 4.65–15.35) [34]. Given this, additional models were conducted with asymptotically distribution-free (ADF) estimation (also known as weighted least squares). ADF does not require normality but studies have shown it is only useful for relatively simple models with a moderate to large sample size (some suggest n > 1000) [53–55]. Results were similar to ML estimation but the difference in CFI between the metric model and structural model was -.012. The majority of studies of invariance testing procedures have been undertaken using ML estimation. Also, there are no standards on appropriate tests and cut-offs for alternative estimation methods. There is some indication that ΔRMSEA performs well with ordinal data [56, 57].
